# Circulating DNA: Diagnostic Tool and Predictive Marker for Overall Survival of NSCLC Patients

**DOI:** 10.1371/journal.pone.0038559

**Published:** 2012-06-12

**Authors:** Raquel Catarino, Ana Coelho, António Araújo, Mónica Gomes, Augusto Nogueira, Carlos Lopes, Rui Medeiros

**Affiliations:** 1 Molecular Oncology Group Investigation Center, Portuguese Institute of Oncology, Porto, Portugal; 2 Medical Oncology Department, Portuguese Institute of Oncology, Porto, Portugal; 3 Abel Salazar Institute for the Biomedical Sciences, Porto, Portugal; 4 Research Department, Portuguese League Against Cancer (Norte), Porto, Portugal; 5 Faculty of Health Sciences of Fernando Pessoa University, CEBIMED, Porto, Portugal; Johns Hopkins University, United States of America

## Abstract

**Purpose:**

The purpose of our study was to determine whether the amounts of circulating DNA (cDNA) could discriminate between NSCLC patients and healthy individuals and assess its value as a prognostic marker of this disease.

**Methods:**

We conducted a study of 309 individuals and the cDNA levels were assessed through real-time PCR methodology.

**Results:**

We found increased cDNA levels in NSCLC patients compared to control individuals. We also found a decreased overall survival time in patients presenting high cDNA levels, when compared to lower cDNA concentrations.

**Conclusions:**

Quantification of cDNA may be a good tool for NSCLC detection with potential for clinical applicability.

## Introduction

Lung cancer is a worldwide problem. At the time of diagnosis, 50% of patients have advanced incurable disease. Different chemotherapy combinations – with or without targeted therapies – yield similar results despite the continuous efforts of clinicians [1].

Non-small cell lung cancer (NSCLC) accounts for about 80% of lung cancers. No more than 10% of these can be treated with surgery because of the lack of an early diagnosis or because of a high frequency of metastasis at diagnosis [2]. The prognosis is poor with only 10–15% of patients surviving 5 years after diagnosis. This dismal prognosis is attributed to the lack of efficient diagnostic methods for early detection and lack of successful treatment for metastatic disease. Within the last decade, rapid advances in molecular biology and radiology have provided a rational basis for improving early detection and patients’ outcome. The aim for early detection is to identify lung cancer at a stage early enough to be curable by surgery. Prognostic factors predict survival independent of the treatment applied and can classify patients as high or low risk. The most important prognostic factor is stage according to the TNM system [3], [4]. Other prognostic factors include clinical aspects, as gender, age, weight loss and cardiovascular disease, elevated lactate dehydrogenase levels, FDG-PET scan and pathological aspects [5], [6]. A major hurdle in the attempts to improve the survival of these patients has been the lack of a simple, non-invasive and effective test for early prediction of therapeutic efficacy.

The finding that tumors are capable of shedding DNA into the blood stream, which can be recovered from both serum and plasma and used as surrogate source of tumor DNA, has opened new areas in cancer diagnosis and prognosis [7]. A number of studies have examined the mechanism behind origin and release of free DNA in the circulation and its clinical implications for lung cancer diagnosis, prognosis, and monitoring the effect of cytotoxic therapies [7].

The presence of extracellular nucleic acids in the human bloodstream was first described in 1948 by Mandel and Métais [8]. In recent years, many studies identified genetic alterations in cell-free circulating DNA (cDNA) from plasma or serum and tumor DNA in many tumor types, including lung cancer [9].

Previous studies indicate that circulating nucleic acids in peripheral blood are originated from tumors, through apoptosis, necrosis or cell lysis of tumor cells [10], [11]. The presence of tumor DNA in blood is probably the result, in variable proportions, of these different mechanisms, such as apoptosis, necrosis, cell lysis and circulating tumor cells lysis, which produce DNA leakage or excretion.

There are multiple potential uses for cDNA quantification in cancer diagnosis and prognosis. It may represent a valuable source of tumor DNA when the exact position of a suspected primary lesion is not clearly defined, or when biopsies are not available [12]. However, direct comparison of the available data is often prevented by differences in the parameters analysed and by lack of standardized methodology and analysis procedures. Any future application of plasma or serum DNA analysis for diagnostic purposes will depend on the reproducibility and reliability of results, both of which require optimization and equivalent procedures. For this study, we selected an assay designed for the human telomerase reverse transcriptase (*hTERT*) genomic sequence that was consistently developed in a previous study [13]. The quantification of circulating DNA through a sophisticated molecular method can be a useful diagnostic tool to differentiate patients with tumors from healthy individuals and can identify those with increased risk of cancer. There are several studies that have valued the different concentration of free plasma DNA among patients with lung tumors and healthy individuals, and have used different data analysis methods and laboratory procedures [14]. So far, the different methods utilized for sample processing and storage, for the extraction and quantification of plasma DNA and the choice of different target genes, have not allowed the use of circulating DNA in the clinical practice [15]. The evaluation of the reproducibility of data obtained from other groups is necessary for any eventual future application of this test. For this purpose, we decided to apply a real-time PCR methodology previously described [16], in order to confirm the reproducibility of the results.

For this study, we selected an assay designed for the human telomerase reverse transcriptase genomic sequence. Amplification of *hTERT* was therefore used as a marker of the total amount of DNA present in plasma samples. *hTERT* expression and telomerase activity have already been reported as prognostic factors in NSCLC patients [17], [18] **.** However, this study is not focused in the evaluation of *hTERT* expression as a tumor-associated marker. Instead, our working hypothesis was based on the use of a single copy gene, such as *hTERT,* as an indicator of the global amount of circulating DNA. This methodology has already been described by Sozzi and co-workers, and we processed our samples using the same analytic and pre-analytic methods that have been reported [16].

The present study was designed to analyze the efficacy of circulating plasma DNA levels in discriminating lung cancer patients from healthy individuals. Additionally, the utility of circulating plasma DNA as a prognostic marker of survival and in predicting response to therapy and disease progression was also assessed.

## Materials and Methods

### Study Population

We evaluated 104 newly diagnosed and untreated patients with non-small lung cancer (NSCLC) and 205 control individuals. Since 2002, 104 consecutive Caucasian patients admitted to the Portuguese Institute of Oncology of Porto (IPO-Porto), Portugal, with cytological or histological confirmed NSCLC, have been prospectively recruited to the study (median age 64.0 years; mean age 63.2 years; sd 10.0).

Considering the patientś gender, 20 (18.9%) were female and 86 (81.1%) male individuals and regarding smoking habits, 24 (23.1%) were non-smokers and 80 (76.9%) were smoker or former-smoker individuals.

The control group consisted of 205 healthy individuals, 78 (38.0%) male and 127 (62.0%) female individuals, with a median age of 50.0 years (mean age 47.3; standard deviation 11.6), without clinical history of cancer, from the same geographic area as the case group.

The patients were evaluated according to the TNM staging system, and the assessment of tumour response to chemotherapy was based on RECIST (Response Evaluation Criteria In Solid Tumors). The first line chemotherapeutic protocol consisted of platin-based doublet chemotherapy in combination with a third-generation cytotoxic compound such as paclitaxel, gemcitabine or docetaxel. The chemotherapeutic protocols were as follows: cisplatin (80 mg/m2 on day 1) + paclitaxel (175 mg/m2 on day 1 every 3 weeks); cisplatin (100 mg/m2 on day 1) + gemcitabine (1250 mg/m2 on days 1 and 8 every 3 weeks); carboplatin (AUC 6 on day 1) + paclitaxel (175 mg/m2 on day 1 every 3 weeks); carboplatin (AUC 6 on day 1) + gemcitabine (1000 mg/m2 on days 1 and 8 every 3 weeks).

The median follow-up time was 9 months (range 1–35 months). Patients’ distribution according to the stage at the time of diagnosis was 4 patients (3.8%) presenting localized disease (stages I and II) and 100 (96.2%) with advanced disease (stages III and IV). From all patients, the histological type distribution was as follows: 38 patients (36.6%) with epidermoid NSCLC, 54 (51.9%) with NSCLC adenocarcinoma and 12 (11.5%) with other NSCLC histological types.

The study was approved by the institute’s ethics committee. Informed written consent was obtained from all the subjects, according to Helsinki Declaration.

### Sample Collection and DNA Isolation

A 5 ml sample of peripheral blood extracted from each patient was collected in tubes containing EDTA and plasma was immediately separated from the cellular fraction by centrifugation at 2500 rpm for 10 min at 4°C. The resulting supernatant (plasma) was frozen at –80°C. DNA was extracted from plasma by using QIAamp DNA Mini kit (Qiagen, Hilden, Germany) according to the manufacturer’s protocol. The purified DNA from 200 µL of plasma was eluted in a final volume of 70 µL of buffer AE used for the elution and stored at –20°C.

### DNA Quantification in Plasma

The quantification of cDNA was performed by a real-time quantitative PCR method, based on a continuous monitoring of fluorescence by an optical system [19]. The probe is labelled by two fluorescent dyes, one serves as a reporter on 5′ end (VIC dye; Applied Biosystems, Foster City, CA). The emission spectrum of the dye is quenched by a second fluorescent dye at the 3′ end (TAMRA; Applied Biosystems). Primers and probes were designed previously by Sozzi and co-workers to amplify the gene of interest, the *hTERT* single copy gene mapped on 5p15.33 [13]. The primers and probe’s sequences were the following: primer forward, 5′-GGC ACA CGT GGC TTT TCG- 3′; primer reverse, 5′- GGT GAA CCT CGT AAG TTT ATG CAA- 3′; probe, VIC5’- TCA GGA CGT CGA GTG GAC ACG GTG-3′ TAMRA.

Fluorogenic PCRs were carried out in a reaction volume of 25 µL in a 7300 ABIPRISM System (Applied Biosystems). Each PCR reaction consisted of TaqMan Universal Mastermix (Applied Biosystem), probe (7.5 mmol/L), primer forward (5 mmol/L), primer reverse (5 mmol/L) and sterile water. DNA solution (2.5 µL) was used in each real-time PCR reaction. Thermal cycling was performed as previously described [13], and was initiated with a first denaturation step of 50°C for 2 minutes and then 95°C for 10 minutes followed by 50 cycles of denaturation at 95°C for 15 seconds and annealing and extension at 60°C for 1 minute. Each plate consisted of patient samples in duplicates and triplicate blanks of water as negative control. The calibration curve of each plate was calculated based on a dilution series of the TaqMan Control Human Genomic DNA Standard (Applied Biosystems) at 10 ng/ µL: 1, 0.1, 0.01, 0.001 and 0.0001 ng/µL. All the data were analyzed using the 7300 System – SDS Software (version 1.2.3) Sequence Detection Software (Applied Biosystems).

Standard PCR amplification of cyclin D1 gene was performed in order to confirm the real-time PCR results. The protocol was performed according to previously described [20]. Briefly, the PCR reactions consisted of 30 ρmol of each primer, 0.2 mM of each dNTP, 1.5 mM MgCl_2,_ 1 × *Taq* Buffer, and 1 U of *Taq* DNA polymerase to a final volume of 50 µl. Primers used in the analysis were CYF (5′GTG AAG TTC ATT TCC AAT CCG C 3′) and CYR (5′ GGG ACA TCA CCC TCA CCC TCA CTT AC 3′). Thirty five cycles were performed, consisting of an initial heating at 95°C for 10 min to activate the enzyme, followed by 35 cycles of denaturation at 94°C for 1 min, annealing at 55°C for 1 min and extension at 72°C for 1 min, with a final extension step at 72°C for 2 min. PCR products (15 µl) were visualized by electrophoresis on 3% (v/v) agarose gels containing 0.5 µg/ml ethidium bromide. This analysis was performed with 40 edged samples obtained with real-time PCR methodology. Plasma samples of NSCLC patients with the highest and lowest values of cDNA, 20 samples each, were selected for this analysis. The band intensities from standard PCR were compared with the real-time PCR results.

### Statistical Analysis

Data analysis was performed using the computer software SPSS for Windows (version 18.0) (SPSS Inc, Chicago, IL).

Linear amplification down to the last dilution was obtained in each experiment (Pearson correlation coefficient, 0.999 to 0.995).

DNA concentrations between healthy controls and patients were compared using the Mann-Whitney U- test. The Mann-Whitney test was also used to compare DNA levels and patients’ clinical-pathological characteristics. *P*≤0.05 was considered statistically significant. For the diagnostic discrimination of DNA concentrations between cancer patients and controls the area under the curve of the receiver operating characteristic curve (AUC–ROC) was assessed non-parametrically. A *p*-value of ≤0.05 was regarded as significant. An AUC-ROC equal to 1 denotes perfect discrimination between patients with cancer and patients without cancer, a value equal to 0.5 denotes the lack of discrimination, and values in between indicate a degree of discrimination between strong and poor.

Chi-square analysis was used to compare categorical variables and a 5% level of significance was used in the analysis. The odds ratio (OR) and its 95% confidence interval (CI) were calculated as a measurement of the association between the amount of cDNA and probability of lung cancer presence. Logistic regression analysis was used to calculate the adjusted OR (aOR) and 95% CI, with adjustment for age, gender and smoking status.

The probabilities of survival were calculated, and the means and life tables were computed using the product-limit estimate of Kaplan and Meier. The curves were examined by the Log rank test, a statistical test for equality of survival distributions. Survival duration was defined as the time between diagnosis and either death or the last clinical evaluation of the patient. Clinical-pathological data and cause of death was determined from the patient’s medical records.

## Results

### Distributing the CDNA Values between Healthy and NSCLC Patients

Our study was developed to investigate cell free circulating DNA (cDNA) by a real time PCR quantitative approach, performing all the technical procedures necessary for accurate and reproducible results. The quantification of cDNA was performed as previously described, by a real-time quantitative PCR assay [13].

We confirmed the real-time PCR results with standard amplification of cyclin D1 gene. We chose a different marker in order to confirm the relative amount of cDNA in plasma samples from NSCLC patients. Concordance of the amount of cDNA was obtained for all tested samples (data not shown).

This quantitative assay shows a strong discrimination power and a high sensitivity and specificity using the *hTERT* gene as a target sequence for quantification of cDNA in plasma. For patients with two or more samples collected after diagnosis and before treatment, the mean cDNA concentration was calculated.

Plasma DNA was not associated with age and smoking history in both case and control groups. However, considering the control group, cDNA levels were increased in men individuals (Table 1). There were no statistically significant differences in cDNA concentrations between probands in different histological groups and tumor stages (Table 2).

**Figure 1:**
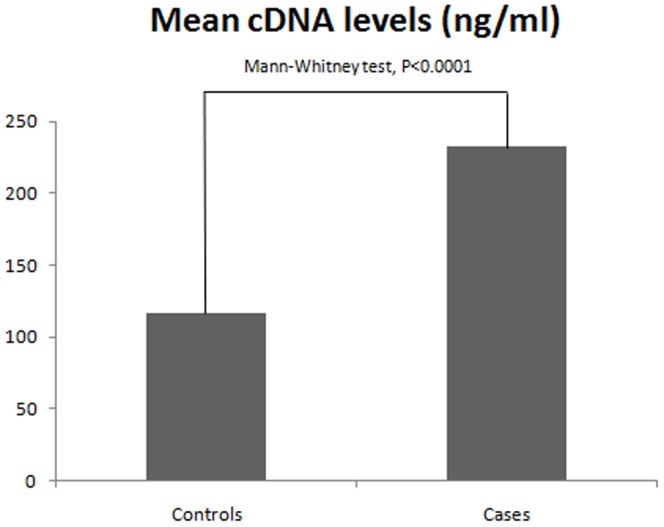
Mean rank of cDNA plasma concentrations in pre-treatment NCLC patients and control individuals. *Mann-Whitney U-test.

The mean cDNA plasma concentration in the control group (mean rank 122.7 ng/ml), was statistically different from the mean cDNA plasma concentration of the patient group (mean rank 270.0 ng/ml) (Mann-Whitney test, p<0.0001) (Figure 1).

**Table 1:** Correlation between circulating plasma DNA levels and demographic characteristics of the subjects.

Characteristics	Mean rank of plasma DNA level (ng/ml)			
	Cases (n = 104)	P* value	Controls (n = 205)	P* value
**Age (years)**				
<64	54.70	0.614	101.48	0.342
≥64	51.68		69.33	
**Gender**				
Female	43.95	0.123	91.57	<0.001
Male	55.72		121.62	
**Smoking history**				
No	50.33	0.688	58.72	0.217
Yes	53.15		66.79	

* Mann-Whitney test.

**Table 2:** Correlation between circulating plasma DNA levels and clinic-pathological parameters of NSCLC patients.

Characteristics	Mean rank of plasma DNA level(ng/ml)	
	Cases (n = 104)	P* value
**Histological type**		
Epidermoid	51.91	0.981
Non-Epidermoid	52.05	
**Tumor stage**		
I/II	64.25	0.427
III/IV	52.03	

* Mann-Whitney test.

### Sensitivity and Specificity for Circulating Plasma DNA Levels as a Diagnostic Marker

**Figure 2:**
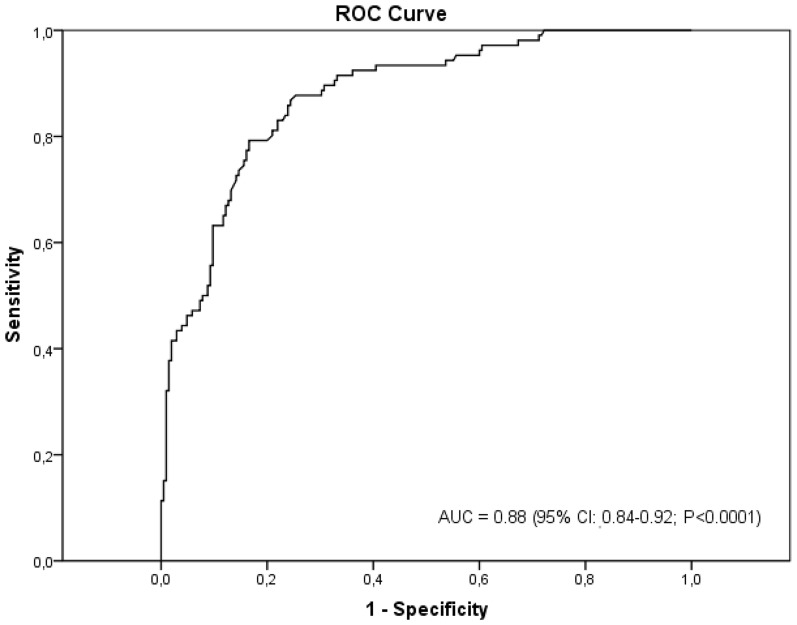
Receiver-operating characteristics (ROC) curve to calculate sensitivity and specificity of circulating plasma DNA as a tumor marker of NSCLC.

The ROC curve was made to evaluate the diagnostic power of the circulating DNA concentration. The ROC analysis is performed through the study of the function that links the sensibility with 1-specificity. The subtended area by the ROC curve (Figure 2) shows a synthetic index of the overall capacity of the test in differentiating between healthy and ill individuals. The closer the area gets to the unit, the greater its discriminating ability. According to our data, the area under the ROC curve is 0.88 (95% CI, 0.84–0.92; p<0.0001). We found that lower cut-off values increased the sensitivity of the assay but at the cost of specificity and vice versa. Using the results of the ROC curve, an analysis was made on the test performance with respect to the different threshold values (Table 3). The results showed that, with a threshold of 20 ng/mL, there is a probability of illness of 71% when the test is positive (PPV). A DNA cut-off level of >20 ng/ml differentiated between lung cancer patients and controls with a specificity of 83% and sensitivity of 79%.

### CDNA Concentration and Risk of NSCLC

An elevated concentration of plasma cDNA was associated with a higher chance for the presence of NSCLC. Our results indicate that high cDNA concentrations are more prevalent among cancer cases, comparing to control individuals (Table 4). Data analysis demonstrates that individuals with high cDNA concentrations present an increased chance for the presence of NSCLC.

**Table 3:** Test performance.

Cut-off value (ng/ml)	Sensitivity	Specificity	PPV	NPV
**3**	100.0	25.85	41.08	100.0
**6**	93.40	50.73	49.50	93.69
**9**	92.45	63.90	56.98	94.24
**12**	89.62	68.78	59.75	92.76
**15**	87.74	74.63	64.14	92.17
**20**	79.24	83.41	71.19	88.60

Keeping the cut-off value as the reference value equal to 6 ng/mL, the relative risks of illness by DNA classes are shown in Table 4. The risk of pathology increases as the DNA concentrations increase: with concentrations between 6.0 and 20.0 ng/mL the risk for illness is over 3 times higher, when compared with cDNA concentrations lower than 6 ng/mL (OR, 3.33; 95% CI, 1.29–8.58; p = 0.010); high concentrations greater than 20.0 ng/mL are associated with a risk of illness of over 36 times higher (OR, 36.71; 95% CI, 15.49–86.99; p<0.0001). For the cut-off value of 20 ng/ml, logistic regression analysis adjusted by age, gender and smoking status confirmed these associations (aOR, 12.69; 95% CI, 4.95–32.52; p<0.0001).

### CDNA as a Prognostic Marker of NSCLC

We looked at correlation between pre-treatment circulating plasma DNA levels and survival time in 73 NSCLC patients, who received a first line chemotherapeutic protocol consisting of platin-based doublet chemotherapy in combination with a third-generation cytotoxic compound such as paclitaxel, gemcitabine or docetaxel and for which survival data was available. Survival analysis was performed according to plasma DNA levels distribution using the Kaplan–Meier method and Cox-regression analysis.

**Table 4:** Frequency and *odds ratio* of plasma DNA concentrations among the control group and NSCLC patients.

cDNA levels (ng/ml)	Controls (n = 205) N (%)	Patients (n = 104) N (%)	OR*	95% CI*	P*
<**6**	104 (93.7)	7 (76.3)	1.00	Reference	-
[**6]**–[**20**]	67 (81.7)	15 (18.3)	3.33	1.29–8.58	0.010
≥**20**	34 (28.8)	84 (71.2)	36.71	15.49–86.99	<0.0001
<**20**	171 (88.6)	22 (11.4)	1.00	Reference	-
≥**20**	34 (28.8)	84 (71.2)	19.20	10.58–34.87	<0.0001

* For 20 ng/ml cut-off value, P<0.0001, OR = 12.69 and 95% CI: 4.95–32.52, using logistic regression analysis adjusted by age, gender and smoking status.

**Figure 3:**
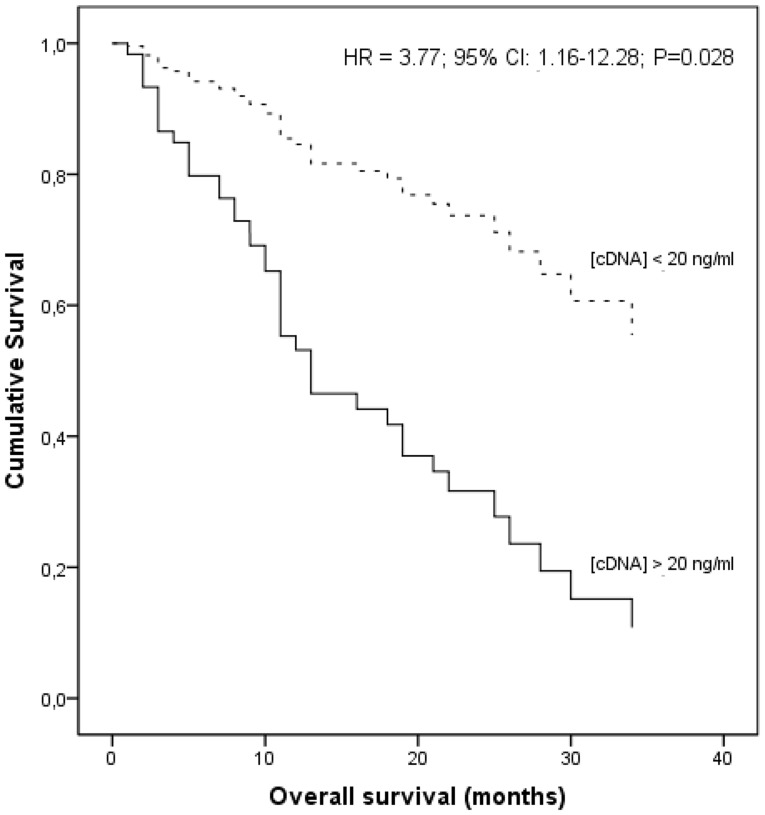
Cox regression analysis of overall survival according to cDNA levels, with tumor stage as covariate.

The mean survival rates were statistically different according to the patients’ cDNA concentrations, indicating that patients with high cDNA levels presented a lower mean survival time than the other patients (16.8 vs 22.4 months; Log Rank test, p = 0.024). Using a multivariate Cox regression model, we found a decreased overall survival time in patients presenting high cDNA levels (above 20.0 ng/ml), when compared lower cDNA concentrations, with stage (P = 0.171) as covariate (hazard ratio, HR = 3.77; 95% CI: 1.16–12.28; *P* = 0.028) (Figure 3).

## Discussion

Diagnostic assays based on blood sample analysis are becoming an area of study with growing interest, mainly because of the simplicity of sampling and the future potential of automation of the technical methods for clinical applicability. The presence of circulating tumor DNA in plasma of patients with lung cancer arouse great interest since, with a simple blood test, a valid marker could be set out for a possible screening, diagnosis, prognosis, progression of disease and the monitoring of treatment response. Unfortunately, the standardization of the test is difficult because of the lack of a suitable standard test and the use of different extraction and quantification methods [14], [21].

The presence of abnormally high levels of free circulating DNA in plasma/serum of cancer patients was first demonstrated in 1977 [22]. The possibility of recovering tumor-derived DNA from the patient’s blood has provoked great expectation. This method offers a non-invasive means to obtain tumor surrogate material, which could represent a unique source for diagnostic and prognostic applications. However, only recently cell free DNA is becoming an issue of growing interest and its possible use as a marker for cancer diagnosis or prognosis has been investigated. Recent studies demonstrated higher cDNA levels in lung cancer patients comparing to control individuals, however, the laboratorial methods have not been consistently evaluated, namely the procedures for DNA isolation and quantification [23]. Moreover, there are some contradictory results regarding the analyses of the clinical-pathological features, such as clinical staging and the prognostic and predictive value of quantification of cell free DNA and relapse-free evaluation that should be studied in more detail [7], [11], [24]. A number of methods have been described to assess the amount of DNA present in plasma, including competitive binding radioimmunoassay, SYBER green, PicoGreen and others, but most lack the sensitivity and specificity required.

Recent studies used real-time PCR methodology for cDNA quantification [11], [23], [25], [26]. The quantification of a single copy gene seems a sensitive and adequate method for assessing cDNA levels, and various markers have already been tested, namely, amplification of hTERT [13], [26], lactate dehydrogenase [11] and beta-globin [25] genes. In the present work, we used real-time PCR for DNA quantification, which can be regarded as the standard method currently available [26].

In our study, we found no statistically significant differences in cDNA concentrations between probands in different histological groups and tumor stages. However, there is some controversy in the relationship between DNA levels and the clinical-pathological features [27]. Conflicting data have also been reported about circulating DNA as a prognostic factor in lung cancer patients. Some studies showed a correlation between an elevated plasma DNA concentration and poor survival [11], [25], whereas other studies did not report such a relationship [16], [26]. This might be explained by differences in patient selection, covering both non-small cell lung cancer (NSCLC) and small cell lung cancer (SCLC), and both treated and untreated patients. Moreover, techniques for sample collection and DNA quantification differed between these studies. Thus, at present, the prognostic value of circulating DNA for survival has not been established yet. Also, the relationship of circulating DNA with age, gender, histology, stage and pulmonary inflammatory conditions is not clear. Our results are in agreement with the observations found in other studies, regarding the lack of correlation between plasma DNA levels and clinical-pathological parameters [11]. A correlation with age was reported in some [1], [13], but not in all studies [16], [25]. Similarly, no correlation has been established with histologic subtypes [16], [28], [29], [30]. Disparity also exists with regard to variables such as clinical staging. Some studies report that plasma DNA was highest in patients with stage IV disease [29], whereas in other studies there was no such association [11], [13], [16], [22], [28], [30]. As already discussed, these discrepancies in results across the studies might be due to technical differences prevalent in various studies. These differences provide an insight into the present status of circulating DNA research and of the need for larger controlled studies with standardized procedures.

We found increased levels of circulating DNA in NSCLC patients compared to control individuals. Similar results were also found in other studies, with higher DNA plasma concentrations found in cancer patients when compared to healthy individuals [13], [16], [26], [31], [32], [33]. According to the majority of the quantitative studies performed until the present time, cell free circulating DNA is observed in healthy subjects at concentrations between 0 and 100 ng/ml of blood with an average of 30 ng/ml, whereas in cancer patients the concentration in plasma or in serum varies between 0 and 1000 ng/ml, with an average of 180 ng/ml [12]. However, these values constitute just a reference to evaluate cell free DNA concentrations, as the percentage of circulating DNA originated from tumor cells can vary from one cancer model to another [12]. The plasma DNA concentrations observed in this study were similar to the cell free DNA level averages described in the literature [1], [11], [16], [34].

The presence of free circulating DNA can be found in patients with malignant pathologies, but also in healthy individuals and in patients with non-malignant diseases, namely erythematic lupus, rheumatoid arthritis, lung embolism, myocardium infarction, traumas or invasive therapeutics practices [35], [36], [37]. However, DNA concentrations seem to be much higher in patients with malignant pathologies [27], [37]. There are various advanced hypotheses about the release mechanisms of DNA in circulation. For healthy individuals, it is presumed that circulating DNA originates from the death of lymphocytes or other nucleated cells, whereas for patients with neoplastic pathologies, there were several hypothesis, namely lysis of tumor cells [12].

The purpose of our study was to confirm the results of the previous researches about the free circulating DNA’s capacity of differentiating healthy individuals from patients with NSCLC cancer and to validate the method used for future clinical application.

Increased plasma cDNA concentration was associated with a higher probability of lung cancer presence. To our knowledge, similar OR values were never reported previously for any biologic marker and could be of substantial benefit in clinical practice. Our study has confirmed the literature data, indicating that higher DNA concentrations can be correlated with the condition of the disease.

High plasma DNA levels were also associated with decreased overall survival. Although there are conflicting results [31], some studies also found a correlation between an elevated plasma DNA concentration and poor survival [11], [25], [26], [32].

A recent study has demonstrated that the levels of cell free DNA present in cancer patients constitute a stable parameter over time and its variations may be due to real clinical alterations in the patient, as long as all the technical and methodological steps are controlled [38]. Therefore, it becomes important to carefully monitor the methods for blood sampling, plasma DNA isolation and DNA quantification. Some quantification methods, such as colorimetric kits, are not based on the amount of amplifiable DNA but in the total amount of nucleic acids, which can include double-stranded and single-stranded DNA and RNA.

A different approach has been described by Silva and co-workers in colon cancer [39]. This study investigated the presence of RNA from epithelial tumors in plasma from patients with colorectal carcinomas and its correlation with tumor characteristics and circulating tumor cells. Epithelial tumor RNA was associated with advanced stages and circulating tumor cells.

The differences of the results observed in literature may also reflect biological causes (histology, tumor origin, stage or tumor size) or technical issues (blood processing, cell free DNA isolation and quantification). There is insufficient data for the comparison of all these parameters between different types of cancer, as it is still unclear, whether all types of cancer can release altered cell free DNA and at the same rate [12]. It has been demonstrated that clearance of cell free DNA from the bloodstream seems to be a rapid process, and one possible explanation is based on the cell free DNA sensibility to plasma nucleases, such as DNAse1 [40]. Moreover, a study has demonstrated that, for a patient with a tumor load of 100 g in size, up to 3.3% of the tumor DNA entered the circulation every day [41]. In another study, mutations in *TP53* or *KRAS2* were detected in whole blood at least one year before diagnosis in 67% of patients, which indicates that tumor DNA is released in blood before conventional diagnosis and enhances the importance of this marker in cancer prevention [42].

Other tumor markers that play an important role in lung carcinogenesis and are implicated in the treatment response of NSCLC patients should also be evaluated, namely cyclin D1, kRAS and EGFR (epidermal growth factor receptor). These markers have been intensively studied in oncology and are directly associated with lung cancer etiology. The analysis of these markers should be performed in future works, in order to clarify its correlation with lung cancer phenotype, tumor aggressiveness and circulating DNA.

We think that the results are promising enough to encourage further research in the area of circulating DNA as a tool for monitoring therapeutic efficacy in lung cancer patients. Identification of additional, more specific, and more sensitive plasma-based biomarkers, which can be used in combination with circulating DNA, may further improve the diagnostic power of current imaging tools for indicating therapeutic efficacy.

## Conclusions

This study demonstrates that higher levels of free circulating DNA can be detected in patients with lung cancer compared to healthy individuals by a quantitative PCR assay. We have shown that the analytic procedure reported by Sozzi et al [13] is useful to discriminate between healthy subjects and NSCLC patients. The reproducibility of this method could represent a first step for its future application in the clinical practice.

Although some concerns regarding the sample size and age of case and control groups may be evaluated, this study suggests a non-invasive diagnostic tool that can be further investigated in future prospective studies for detection of lung cancer and assessment of its potential applicability as a complementary diagnostic and prognostic tool in clinical practice. We believe that our results may help to improve discussion between researchers in the field and that different methodological approaches must be considered regarding the real meaning of cDNA in lung cancer patients.

Levels of circulating DNA could also identify higher-risk individuals for this disease screening and chemoprevention trials. Furthermore, plasma DNA could also be used to detect cancer specific molecular markers, such as mutations and amplifications, and individualize and monitor drug treatment, namely resistance to targeted therapies without the need for repeated tumor biopsies in metastatic disease.

We found a significant decrease in the overall survival for higher concentrations of cell free circulating DNA and a very significant increase in the patients’ risk for the presence of lung cancer in individuals with high cDNA levels. Cell free circulating DNA may represent an important source of biomarkers at several steps of carcinogenesis, including early detection of preneoplastic lesions and monitoring of cancer. Moreover, levels of plasma DNA could be tested as a potential intermediate biomarker of the efficacy of intervention.

Large-scale prospective studies are necessary for population-based studies and molecular epidemiologic studies, in order to implement a clinical application in lung cancer detection, diagnosis, prognosis and prediction of treatment response.
